# Genetic diversity of *Clostridium perfringens *type A isolates from animals, food poisoning outbreaks and sludge

**DOI:** 10.1186/1471-2180-6-47

**Published:** 2006-05-31

**Authors:** Anders Johansson, Anna Aspan, Elisabeth Bagge, Viveca Båverud, Björn E Engström, Karl-Erik Johansson

**Affiliations:** 1 National Veterinary Institute, SE-751 89 Uppsala, Sweden; 2 Division of Bacteriology and Food Hygiene, Department of Biomedical Sciences and Veterinary Public Health, Swedish University of Agricultural Sciences, Uppsala, Sweden

## Abstract

**Background:**

*Clostridium perfringens*, a serious pathogen, causes enteric diseases in domestic animals and food poisoning in humans. The epidemiological relationship between *C. perfringens *isolates from the same source has previously been investigated chiefly by pulsed-field gel electrophoresis (PFGE). In this study the genetic diversity of *C. perfringens *isolated from various animals, from food poisoning outbreaks and from sludge was investigated.

**Results:**

We used PFGE to examine the genetic diversity of 95 *C. perfringens *type A isolates from eight different sources. The isolates were also examined for the presence of the beta2 toxin gene (*cpb2*) and the enterotoxin gene (*cpe*). The *cpb2 *gene from the 28 *cpb2*-positive isolates was also partially sequenced (519 bp, corresponding to positions 188 to 706 in the consensus *cpb2 *sequence). The results of PFGE revealed a wide genetic diversity among the *C. perfringens *type A isolates. The genetic relatedness of the isolates ranged from 58 to 100% and 56 distinct PFGE types were identified. Almost all clusters with similar patterns comprised isolates with a known epidemiological correlation.

Most of the isolates from pig, horse and sheep carried the *cpb2 *gene. All isolates originating from food poisoning outbreaks carried the *cpe *gene and three of these also carried *cpb2*. Two evolutionary different populations were identified by sequence analysis of the partially sequenced *cpb2 *genes from our study and *cpb2 *sequences previously deposited in GenBank.

**Conclusion:**

As revealed by PFGE, there was a wide genetic diversity among *C. perfringens *isolates from different sources. Epidemiologically related isolates showed a high genetic similarity, as expected, while isolates with no obvious epidemiological relationship expressed a lesser degree of genetic similarity. The wide diversity revealed by PFGE was not reflected in the 16S rRNA sequences, which had a considerable degree of sequence similarity. Sequence comparison of the partially sequenced *cpb2 *gene revealed two genetically different populations. This is to our knowledge the first study in which the genetic diversity of *C. perfringens *isolates both from different animals species, from food poisoning outbreaks and from sludge has been investigated.

## Background

*Clostridium perfringens*, an anaerobic Gram-positive bacterium known to be a common pathogen in humans, in domestic animals and in wildlife, is the primary cause of clostridial enteric disease in domestic animals. The complete genome sequence of *C. perfringens *has been published previously [1]. *C. perfringens *has 10 rRNA operons whose heterogeneity was investigated by Shimizu *et al*. [2]. They found 18 polymorphic sites among the 16S rRNA genes. A common feature of *C. perfringens *is the large number of exotoxins produced; 17 different exotoxins have been described in the literature [3]. In addition, *C. perfringens *produces an enterotoxin, CPE [4].

*Clostridium perfringens *is subdivided into five toxinotypes (A – E) based on the production of the four major exotoxins (viz. alpha, beta, epsilon, and iota). The major toxins together with the enterotoxin and the beta2 toxin [5], play an important role in several serious diseases [3,6]. CPE causes food-borne disease in humans, canine enteritis and porcine enteritis. Beta2 toxin, recently described [5], has been associated with enteric diseases in domestic animals, especially piglets [7-9] and horses [10]. However, two recently published studies, by Jost *et al*. [11] and Vilei *et al*. [12], demonstrated that beta2 toxin, encoded by *cpb2*, was expressed by most porcine *C. perfringens *isolates, but seldom by isolates of non-porcine origin. The results of those studies indicate that beta2 toxin does not cause enteritis in animal species other than pigs. Vilei *et al*. [12] reported that gentamicin and streptomycin induced expression of an atypical *cpb2 *gene in a non-porcine isolate. In a recent publication by Waters *et al*. [13], it was reported that *cpb2 *of *C. perfringens *from horses was transcriptionally active and that the levels of *cpb2 *mRNA were 35-fold lower than a high beta2 toxin producing pig isolate. Isolates originating from humans with gastrointestinal diseases carrying both *cpb2 *and *cpe *have recently been described [14,15].

The epidemiological relationship between *C. perfringens *isolates has previously been investigated primarily by pulsed-field gel electrophoresis (PFGE) and in most of these studies a majority of isolates from food poisoning outbreaks were examined [16-21]. *C. perfringens *isolates originating from poultry have also been investigated previously by PFGE [22-24]. The general conclusions drawn from the previously published articles, concerning both food poisoning outbreaks and animals, is that isolates from the same outbreak have very similar patterns while the genetic diversity is high in non-outbreak isolates and isolates selected randomly [17-19,21-24]. The problem of DNA degradation of certain isolates due to endogenous bacterial nucleases, which are rather common among clostridial isolates, has been discussed elsewhere [16,18,25,26].

The purpose of this study was to compare the genetic relationships of *C. perfringens *type A from eight different sources by PFGE. A further aim was to investigate the distribution of the *cpb2 *and *cpe *genes. The *cpb2 *gene from all *cpb2*-positive isolates was also partially sequenced. In this study a generally wide genetic diversity of *C. perfringens *isolates from eight different sources was found. Furthermore, PFGE clearly distinguished between unrelated isolates of *C.perfringens *and supported a clonal relationship between related isolates. Sequence analysis of the partially sequenced *cpb2 *gene revealed two genetically different populations of the gene.

## Results

### PCR

Multiplex PCR detected only the alpha-toxin gene (*plc*) and all isolates were therefore classified as *C. perfringens *type A. Altogether 28 isolates carried the *cpb2 *gene and 17 carried the *cpe *gene (Table 1). The *cpb2 *gene was found in 6 of the 8 groups studied: pigs (83%), horses (60%), sheep (50%), food poisoning outbreaks (20%), sludge (21%) and poultry (10%). The *cpb2 *gene was not detected in any of the isolates from roedeer or wild birds. The *cpe *gene was found in all isolates originating from food poisoning outbreaks and in one isolate each from horse and roedeer. Both *cpb2 *and *cpe *were found in three isolates originating from outbreaks of food poisoning.

### PFGE

**Figure 1:**
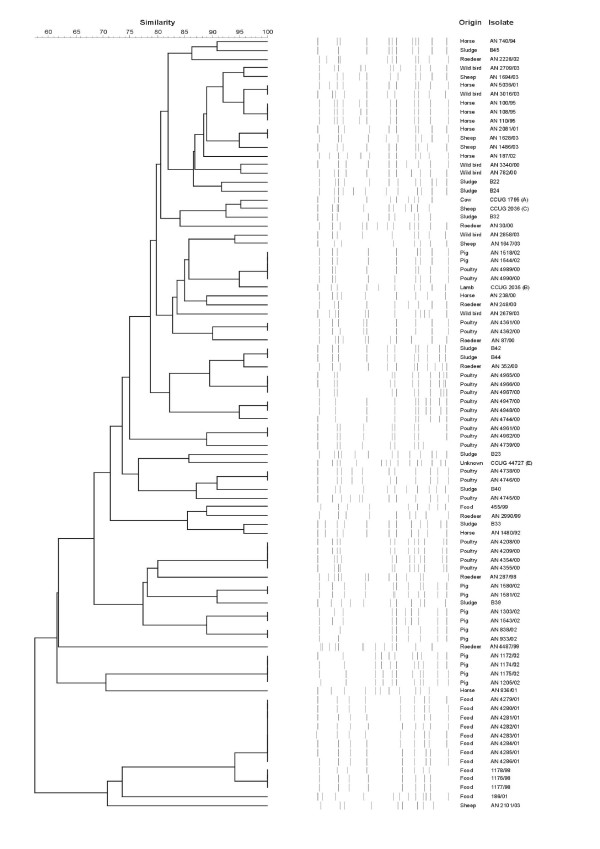
Cluster analysis of *Sma*I-digested DNA from 88 strains of *C. perfringens *separated by PFGE.

**Figure 2:**
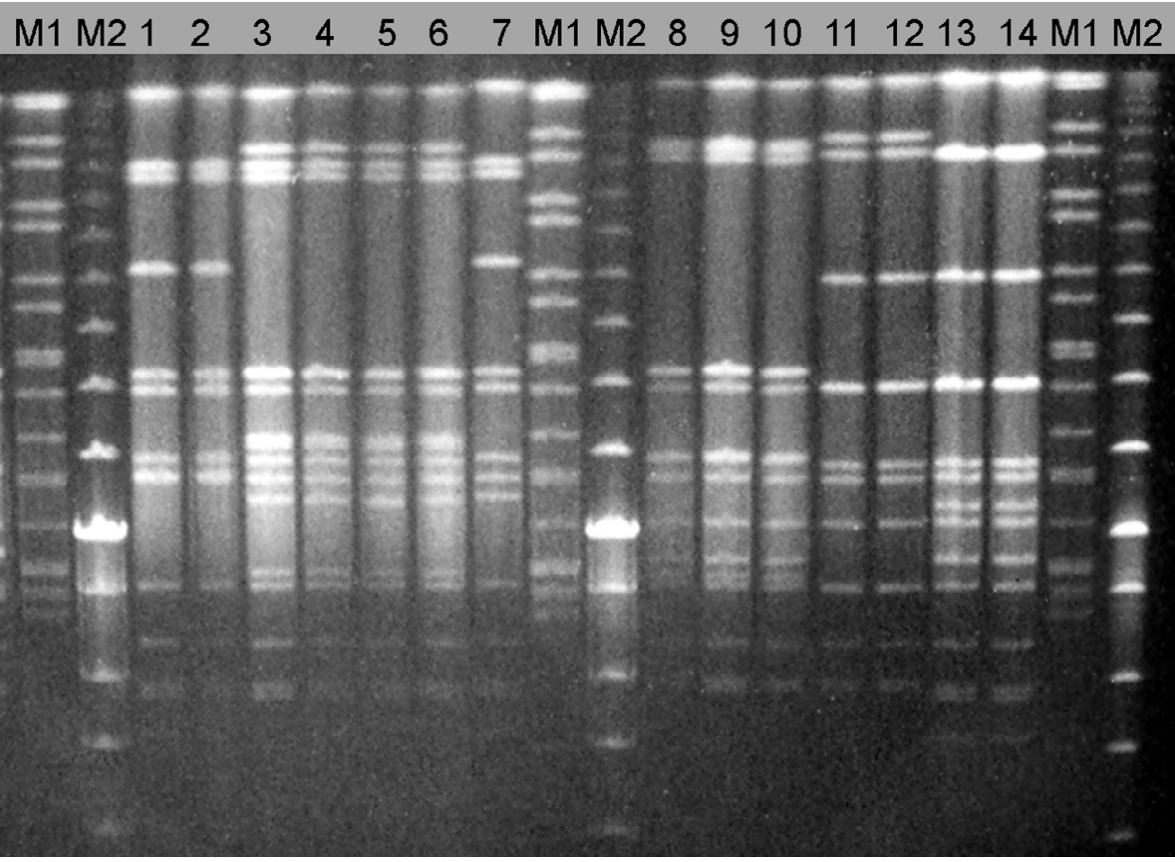
**Representative PFGE patterns of 14 *C. perfringens *isolates**. The patterns obtained by the PFGE protocol with *Sma*I digestion. Lanes M1, chromosomal DNA of *Salmonella *serotype Braenderup (H9812) digested with *Xba*I as reference size markers (size range 33 to 1,135 kb). Lanes M2, lambda ladder with a size range of 0.13 to 194 kb (Low Range PFG Marker, New England Biolabs Inc.). All *C. perfringens *isolates on this gel are from poultry. Lane 1, *C. perfringens *isolate AN 4961/00; lane 2, AN 4962/00; lane 3, AN 4208/00; lane 4, AN 4209/00; lane 5, AN 4354/00; lane 6, AN 4355/00; lane 7, AN 4739/00; lane 8, AN 4965/00; lane 9, AN 4966/00; lane 10, AN 4967/00; lane 11, AN 4989/00; lane12, AN 4990/00; lane13, AN 4947/00 and lane 14, AN 4948/00.

**Figure 3:**
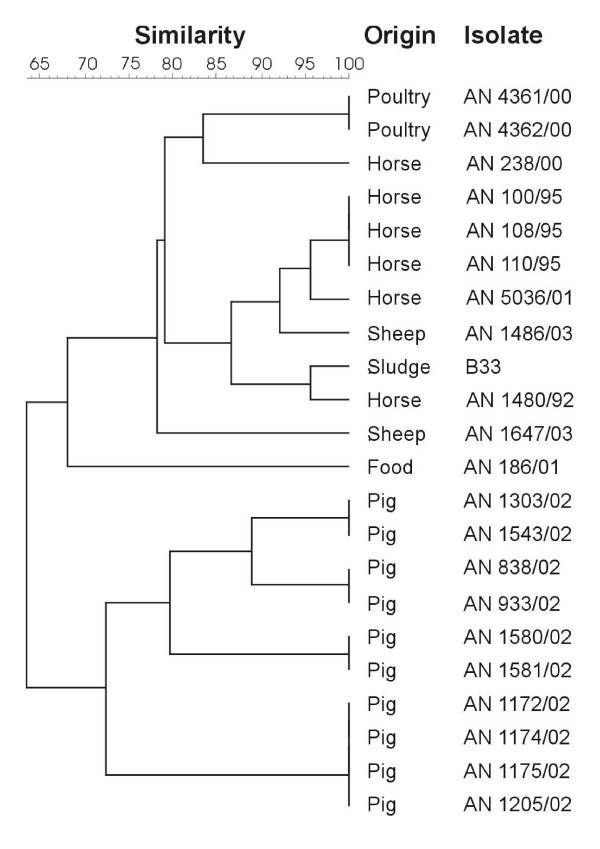
Cluster analysis of *Sma*I-digested DNA from the 22 *cpb2*-positive isolates of *C. perfringens*.

Of the 101 isolates of *C. perfringens *examined in this study, 88 were successfully characterized by PFGE after genomic DNA digestion with *Sma*I (Table 1, Figure 1). The *Sma*I PFGE patterns of some of these isolates are shown in Figure 2. The genetic relatedness of the isolates ranged from 57 to 100% and 56 distinct PFGE profiles were observed. Cluster analysis of PFGE data showed no apparent relationship between the source of the isolate and its PFGE profile. However, the isolates originating from food poisoning outbreak formed distinct clusters in the dendrogram (Figure 1). Altogether, 17 PFGE profiles with indistinguishable patterns were found. Most of the isolates having PFGE profiles with indistinguishable patterns were from the same outbreak of disease. All of the eight food isolates clustering with indistinguishable patterns originated from the same outbreak of food poisoning. The poultry and porcine isolates clustering with indistinguishable patterns were also isolated from the same outbreaks. Two of the PFGE profiles with indistinguishable patterns contained isolates with no obvious epidemiological correlation. Of the 28 *cpb2*-positive isolates, 22 were successfully characterized by PFGE. The *Sma*I PFGE patterns of these isolates are shown in Figure 3. The overall genetic relatedness of the *cpb2*-positive isolates was 63%. All the 10 isolates originating from pigs cluster together with a genetic relatedness of 72%. This cluster was subdivided into four subclusters with 100% genetic relatedness in each cluster.

### Sequence data analysis

**Figure 4:**
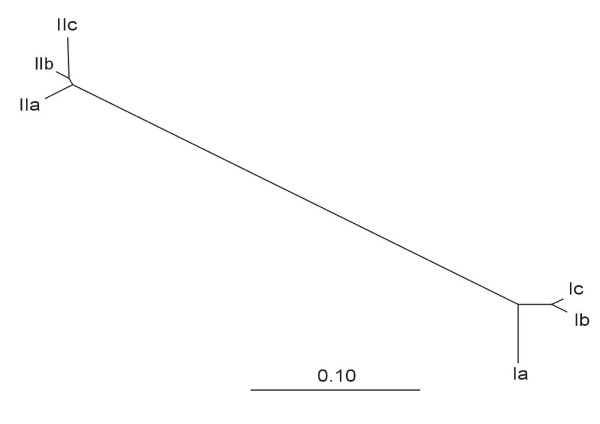
**Radial representation of a phylogenetic tree prepared by the neighbour-joining method showing phylogeny of the *cpb2 *gene**. The length of the scalebar represents 10 nucleotide substitutions per 100 positions.

In the phylogenetic tree (Figure 4) two main groups of the partially sequenced *cpb2 *gene (the distance matrix comprised 425 nucleotide positions, corresponding to positions 282 to 706 in the consensus *cpb2 *sequence) were observed, I and II, each subdivided to three subclusters, a, b, c. A total of six groups could be identified (Figure 4, Table 2). Groups I and II are mutually related, with a sequence similarity of 73.8%. All isolates in our study were found in group I. The sequence similarities within this group varied between 93.3% and 100%. Our *cpb2 *sequences from porcine (Ia) and from non-porcine (Ib) cluster with the *cpb2 *sequences deposited by Vilei *et al *[12]. The three *cpb2 *sequences from food and the horse isolate AN 5036/01 was found in group Ic. Most of the isolates in group II were isolated in the USA, while most of those in group I were isolated in Europe.

Sequencing of the 16S rRNA gene revealed a very high similarity among the 11 isolates analysed. As shown in Table 3, altogether 18 polymorphic sites were detected in the 16S rRNA region between nucleotide no. 91 and 1401 (based on the consensus sequence of the 16S rRNA gene in the 10 rRNA operons of *C. perfringens *strain 13, *rrnA *– *rrnI*). Seven of the polymorphisms found in our isolates were also found in *C. perfringens *strain 13 [2]. At position 154 of the 16S rRNA genes, one nucleotide difference was observed; at this position, isolates from food poisoning outbreaks (AN 4279/01 455/99) had a T, whereas all others had a C. This was the only nucleotide difference involving all operons that was observed in the sequenced isolates.

## Discussion

### Prevalence of the *cpb2 *and *cpe *genes

The high prevalence of the *cpb2 *gene in isolates from pigs and horses is consistent with other studies reporting a high prevalence of *cpb2 *in pigs and horses suffering from gastrointestinal diseases [7-10]. The distribution of *cpe *in isolates of animal origin is low, which also tallies with data from other studies [27-29]. Isolates from food poisoning outbreaks typically carry a chromosomal *cpe *gene [21,30,31]; in this study three of these isolates also carried the *cpb2 *gene. In this study three of the isolates from food poisoning outbreaks also carried the *cpb2 *gene. Isolates originating from humans with gastrointestinal diseases, carrying both *cpb2 *and *cpe*, have been described recently [14,15].

### PFGE analysis

The aim of this study was to elucidate the general genetic diversity of *C. perfringens *type A isolated from a variety of sources in Sweden and Norway. In the present study, any band difference between two PFGE types was considered sufficient to distinguish between two different PFGE types. The results obtained by PFGE reveal a wide genetic diversity and no definite relationship between the source of the isolate and the positions in the dendrogram can be established.

The wide genetic diversity revealed in this study was not unexpected, because of the wide diversity of the isolates analysed. Previous studies on *C. perfringens *by PFGE have shown that isolates from the same food poisoning outbreak have a very similar pattern [18-20] and that *C. perfringens *isolated from retail food showed a high genetic variation [17]. This has also been observed in poultry where isolates from diseased birds showed a similar pattern, whereas the genetic diversity was considerable in isolates from healthy birds [22-24]. Those observations are consistent with our results, where we can see that PFGE profiles with indistinguishable patterns in almost all cases contain isolates having a known epidemiological connection. Comparison of the PFGE patterns of the *cpb2*-positive isolates revealed a clonal relationship among the porcine isolates (group Ia), which cluster together in the dendrogram (Figure 3). However, no clonal relationship could be established for isolates belonging to groups Ib and Ic. The *cpb2 *gene is known to be located on several low copy number plasmids [5,15]. The genetic diversity of *cpb2*-positive isolates is therefore, not surprising, as PFGE mainly reflects the chromosomal diversity.

In this study 13% of the isolates were non-typable due to DNA degradation. This problem has been reported previously [16,18,32]. It is a disadvantage when PFGE is used as a subtyping method for clostridial species. However, problems with DNA degradation have not been reported previously when analysing poultry isolates by PFGE [22-24]. As in this study none of the isolates from poultry, horses or pigs were found degraded, PFGE is a very suitable method for epidemiological investigation of enteric diseases caused by *C. perfringens*. The 16S rRNA gene was sequenced for three of the isolates that were found degraded. Neither DNA degradation nor the generally wide genetic diversity was reflected in the 16S rRNA sequences, which were very similar. The use of *Salmonella enterica *subsp. *enterica *serotype Braenderup, (*Salmonella *Braenderup H9812), digested with *Xba*I as a size marker made it easier to normalize tiff images, than by using a commercially available weight marker, thanks to stable levels of DNA in the prepared plugs [33].

In our opinion, PFGE is a reliable and robust method that can be used in combination with epidemiological data to establish *C.perfringens *as the etiological agent whether in food-borne outbreaks or in outbreaks of enteric disease in domestic animals.

### Sequence analysis of the *cpb2 *gene

Sequence analysis of the *cpb2 *gene (Figure 4) indicated the existence of two evolutionary differing populations based on 425 nucleotide positions, corresponding to positions 282 to 706 in the consensus *cpb2 *sequence. A relatively high sequence difference of 26.2% showed that *cpb2 *evolved into two different variants (I and II). The isolates sequenced in this study all belonged to group I and were divided into three sub-clusters: porcine isolates (Ia), animal isolates of non-porcine origin (Ib), and isolates from food poisoning outbreaks (Ic). One of the horse isolates AN 5036/01 clustered together with the food isolates. Most of the group I *cpb2*-positive isolates were of European origin [12], while most *cpb2*-positive isolates from group II had an American origin [11]. However, isolates containing *cpb2 *isolated from humans with a gastrointestinal disease were distributed in both groups (Ic and IIa), irrespective of origin [15]. Groups I and II both contained isolates capable of expressing CPB2 and also isolates that express non-detectable levels of beta2 toxin [11,12,15]. Jost *et al*. [11] identified a *cpb2 *gene that was present in most non-porcine isolates. The *cpb2 *sequences deposited by Jost *et al*. [11] were all affiliated to group II (IIa, IIb or IIc). Isolates from European animals carrying the *cpb2 *gene were all affiliated to group I. It is interesting that none of the *cpb2*-positive isolates from animals in Europe carried the *cpb2 *gene, which are found in group II.

### Sequence analysis of the 16S rRNA gene

The reason for sequencing the 16S rRNA gene was to ascertain whether the diversity found by PFGE analysis was reflected in the 16S rRNA gene. Isolates that were degraded and those found in different clusters in the PFGE dendrogram showed a very high sequence similarity. However, we found 18 positions with polymorphisms among our investigated isolates (Table 3). These polymorphisms are caused by the fact that *C. perfringens *carries 10 rRNA operons and sequence differences exist between them. Some of these polymorphisms were isolate specific. Seven of those found by us were also found by Shimizu *et al*. [2] who analysed all the 10 rRNA operons of *C. perfringens *strain 13. The polymorphisms found in the *C. perfringens *16S rRNA genes reflect certain diversity. In a few other bacterial species polymorphisms have been used for subtyping [34], but due to the 10 rRNA operons in *C. perfringens*, it is more difficult to use the polymorphism pattern for subtyping.

## Conclusion

In this study a wide genetic diversity of *C. perfringens *was found when isolates from eight different sources were analysed by PFGE. Degradation of DNA was observed in 13% of the isolates investigated. The considerable diversity found by PFGE was not reflected in the 16S rRNA sequences, which were very similar. As expected, *C. perfringens *isolates from the same outbreak seemed to be similar genetically, while isolates with no obvious epidemiological connection differed more noticeably. The groups with the highest genetic relatedness were isolates from food-borne outbreaks and pig isolates carrying the *cpb2 *gene. Isolates from the other sources were more widely dispersed in the dendrogram. Two genetically differing populations of the partially sequenced *cpb2 *gene were found by sequence analysis. Furthermore, isolates causing enteric diseases in humans and other animals seem not to have a strong genetic relatedness, based on PFGE analysis. We conclude that PFGE is a reliable and robust method for genotyping of *C. perfringens *isolates.

## Methods

### Bacterial isolates and growth conditions

Altogether 95 isolates of *C. perfringens *type A were obtained from eight different sources: poultry (*n *= 20), pigs (*n *= 12), horses (*n *= 10), sheep (*n *= 6), roedeer (*n *= 12), wild birds (*n *= 6), food poisoning outbreaks (*n *= 15) and environmental samples (*n *= 14) (Table 1). The isolates were collected between 1994 to 2003 (Table 1), all isolates from sludge were collected 2002. One *C. perfringens *type strain CCUG 1795 (A) and five reference strains, CIP 106526 (A), CCUG 2035 (B), CCUG 2036 (C), CCUG 2037 (D) and CCUG 44727 (E), were also included in the study. CCUG strains were obtained from the Culture Collection of the University of Gothenburg, Sweden; the CIP strain was obtained from the Culture Collection of Institute Pasteur, Paris, France. The *C. perfringens *isolates were identified as type A by applying standard biochemical tests and multiplex-PCR [22]. The isolates were stored at -70°C. Thawed isolates were grown on fastidious anaerobe agar (FAA) (LabM, Bury, Lancashire, England) with 10% defibrinated horse blood and incubated in anaerobic jars at 37°C.

### PCR

The four major toxin genes *plc*, *cpb1*, *iap *and *etx *were detected by a modified version of the multiplex PCR assay of Engström *et al*. [22]. Template DNA (2 μl), prepared by the direct lysis method of Herholz *et al*. [10] was added to a 50 μl reaction mixture, with the following reagents: 50 mM KCl, 10 mM Tris-HCl (pH 8.3), 2.5 mM MgCl_2_, 50 nM of each deoxynucleotide, 1 U of AmpliTaq Gold DNA polymerase (Applied Biosystems, Foster City, USA), 50 nM of each *plc *(α-toxin) primer, 25 nM of each *cpb1 *primer, 100 nM of each *etx *primer and 25 nM of each *iap *primer. The thermocycling (incubations for 1 min at 94°C, 55°C and 72°C, respectively, repeated 35 times) was preceded by incubation for 10 min at 94°C. The presence of the CPB2 gene (*cpb2*) and enterotoxin gene (*cpe*) was also determined. *cpb2 *primers (250 nM) from Herholz *et al*. [10] and *cpe *primers (50 nM) from Kadra *et al*. [35] were used in a duplex PCR. The conditions were as in the multiplex PCR, except for the annealing temperature, which was 59°C. The amplicons were analysed by electrophoresis on a 1.5% agarose gel according to standard procedures.

### PFGE

The isolates analysed by PFGE are listed in Table 1. The PFGE protocol of Lukinmaa *et al*. [20] was followed in all essentials. DNA was digested with *Sma*I. Electrophoresis was performed at 6 V/cm with 2.0% AgaroseNA agar (Amersham Biosciences, Uppsala, Sweden) by using the CHEF-DR II system (Bio-Rad Laboratories, Richmond, Calif, USA). Running conditions for *Sma*I-digested DNA were 0.5 to 40 s for 20 h. Lambda ladder with a size range of 0.13 to 194 kb (Low Range PFG Marker, New England Biolabs Inc.) and *Salmonella *Braenderup (H9812) digested with *Xba*I, which is described in greater detail by Hunter *et al*. [33], were used as molecular weight standards. The DNA bands were visualized on a UV transilluminator and Polaroid photographs of the gels were scanned and images in tiff file format were imported into GelCompar II (Applied Maths, Kortrijk, Belgium). Degrees of similarity between isolates were calculated with 1.4% tolerance and 0.5% optimization by applying the band-based Dice similarity coefficient. Clustering analysis was performed with the unweighted pair group method (UPGMA), by average linkages.

### Sequence analysis of the 16S rRNA genes

The 16S rRNA genes of the isolates were amplified with primers [36] suitable for members of the phylum *Firmicutes *(Table 4). The amplicons were used for cycle sequencing with labelled terminators (Big Dye; Applied Biosystems, Foster City, Calif, USA) and with the sequencing primers listed in Table 4. The sequencing products were analysed with an ABI Prism 3100 genetic analyser (Applied Biosystems) and contigs were generated with the Contig Express program included in the Vector NTI Suite (InforMax, Bethesda, Md, USA). The contigs were edited manually with the Genetic Data Environment software [37] before further analysis and deposition in GenBank.

### Sequence analysis of the *cpb2 *genes

The *cpb2 *gene of the 28 *cpb2*-positive isolates was amplified with the β2 primers (250 nM) from Herholz *etal*. [10] and these primers were also used in the cycle sequencing reaction, as described earlier. The *cpb2 *sequences determined in this work were aligned with sequences retrieved from GenBank by the AlignX program in the Vector NTI Suite. The alignment was checked with the Genetic Data Environment software [37]. A phylogenetic tree comprising 88 *cpb2 *sequences was constructed by the neighbour-joining method [38] from a distance matrix that was corrected for multiple substitutions at single locations by the two-parameter method [39]. A representative of cluster Ib was chosen as outgroup. Only one representative for each subgroup was included in the final tree. The distance matrix comprised 425 nucleotide positions, corresponding to positions 282 to 706 in the *cpb2 *sequence of *C. perfringens*, isolate P762/97 [Gen Bank:AJ537531]. Consenus alignments are shown in Additional file 1.

### Nucleotide sequence accession numbers

The sequences of the *cpb2 *gene of the 28 *cpb2*-positive isolates have been deposited in GenBank (National Center for Biotechnology, Bethesda, Md, USA) under accession numbers [GenBank:DQ201544 – GenBank:DQ201571]. The 16S rRNA sequences have also been deposited in GenBank under accession numbers [GenBank:DQ196132 – GenBank:DQ196142].

## Authors' contributions

AJ, KEJ, AA, VB, BEE participitated in the discussions on the study design, the collection of isolates, analysis and interpretation of the data, and in the writing of the manuscript. AJ and AA carried out the analysis and interpretation of PFGE data. Analysis and interpretation of sequence data of the *cpb2 *gene and 16S rRNA gene were carried out by AJ, KEJ and EB. AJ was the principal author of the manuscript. All authors read and approved the final manuscript.

## Supplementary Material

Additional File 1 Consensus alignment of *cpb2 *clusters Click here for file

## Figures and Tables

**Table 1:** The *Clostridium perfringens *type A isolates used in this study

Isolate	Description (Genotype)
**Reference strains**	
CCUG 1795 (A)	*C. perfringens *typestrain, type A (ATCC 13124)
CIP 106526 (A)^b^	*C. perfringens *reference strain, type A
CCUG 2035 (B)^a^	*C. perfringens *reference strain, type B
CCUG 2036 (C)^b^	*C. perfringens *reference strain, type C
CCUG 2037 (D)	*C. perfringens *reference strain, type D
CCUG 44727 (E)^a^	*C. perfringens *reference strain, type E
**Poultry, Sweden**	
AN 4208/00	Cholangiohepatitis, broiler
AN 4209/00	Cholangiohepatitis, broiler
AN 4354/00	Cholangiohepatitis, broiler
AN 4355/00	Cholangiohepatitis, broiler
AN 4361/00	Cholangiohepatitis, broiler (*cpb2*)
AN 4362/00	Cholangiohepatitis, broiler (*cpb2*)
AN 4738/00	Necrotic enteritis, layer
AN 4739/00	Necrotic enteritis, layer
AN 4744/00^a^	Necrotic enteritis, layer
AN 4745/00	Necrotic enteritis, layer
AN 4746/00	Necrotic enteritis, layer
AN 4947/00	Necrotic enteritis, broiler
AN 4948/00	Necrotic enteritis, broiler
AN 4961/00	Cholangiohepatitis, broiler
AN 4962/00	Cholangiohepatitis, broiler
AN 4965/00	Healthy, layer
AN 4966/00	Healthy, broiler
AN 4967/00	Healthy, broiler
AN 4989/00	Cholangiohepatitis, broiler
AN 4990/00	Cholangiohepatitis, broiler
**Horse, Sweden**	
AN 740/94	Healthy (*cpe*)
AN 836/94	Healthy
AN 100/95^c^	1 day post antibiotic treatment with erythromycin (*cpb2*)
AN 108/95^c^	4 days post antibiotic treatment with erythromycin (*cpb2*)
AN 110/95^a,c^	7 days post antibiotic treatment with erythromycin (*cpb2*)
AN 238/00	Diarrhoea (*cpb2*)
AN 2081/01	Diarrhoea
AN 5036/01	Diarrhoea (*cpb2*)
AN 1480/02	Diarrhoea (*cpb2*)
AN 187/02	Diarrhoea
**Pig, Sweden**	
AN 838/02	Enteritis (*cpb2*)
AN 933/02	Enteritis (*cpb2*)
AN 1172/02	Enteritis (*cpb2*)
AN 1174/02	Enteritis (*cpb2*)
AN 1175/02	Enteritis (*cpb2*)
AN 1205/02^a^	Enteritis (*cpb2*)
AN 1303/02	Acute Enteritis (*cpb2*)
AN 1518/02	Acute Enteritis
AN 1543/02	Acute enteritis (*cpb2*)
AN 1544/02	Trauma head
AN 1580/02	Enteritis (*cpb2*)
AN 1581/02	Acute enteritis (*cpb2*)
**Sheep, Sweden**	
AN 1486/03	Enteritis (cpb2)
AN 1628/03	Acute haemorrhagic inflammation
AN 1647/03	Enteritis (*cpb2*)
AN 1694/03	Enteritis
AN 2101/03	Dead animal
AN 2679/02^b^	Enteritis (*cpb2*)
**Roedeer, Sweden**	
AN 3495/96^b^	Enteritis
AN 3496/96^b^	Enteritis
AN 3499/96^b^	Enteritis
AN 287/98	Enteritis
AN 2990/99^a^	Enteritis
AN 4081/99^b^	Enteritis
AN 4487/99	Enteritis
AN 30/00	Enteritis
AN 87/00	Enteritis
AN 351/00	Dead roedeer
AN 248/00	Enteritis (*cpe*)
AN 2226/02	Dead roedeer
**Wild birds, Sweden**	
AN 782/00	Enteritis, Buzzard
AN 3340/00	Enteritis, Herring gull
AN 2679/03	Enteritis Mallard
AN 2709/03	Enteritis and botulism, Mallard
AN 2858/03	Enteritis Herring gull
AN 3016/03	Entertoxaemia, Mallard
**Sludge, Sweden**	
B22^a^	Waste water treatment plant
B23	Waste water treatment plant
B24	Waste water treatment plant
B26^a,b^	Waste water treatment plant
B29^b^	Waste water treatment plant (*cpb2*)
B32	Waste water treatment plant
B33	Waste water treatment plant (*cpb2*)
B34^a,b^	Waste water treatment plant (*cpb2*)
B37^b^	Waste water treatment plant (*cpb2*)
B39	Waste water treatment plant
B40	Waste water treatment plant
B42	Waste water treatment plant
B44	Waste water treatment plant
B45	Waste water treatment plant
**Food**	
1176/98^d^	Norwegian outbreak 3, Meat stew (*cpe*)
1177/98^d^	Norwegian outbreak 3, Meat stew (*cpe*)
1178/98^d^	Norwegian outbreak 3, Meat stew (*cpe*)
455/99^a,d^	Norwegian outbreak 4, Chicken stew (*cpe*)
186/01^d^	Norwegian outbreak 2, Wild meat stew (*cpb2*, *cpe*)
1509/03^b,d^	Norwegian outbreak 1, Meat stew (*cpb2*, *cpe*)
1508/03^b,d^	Norwegian outbreak 1, Meat stew (*cpb2*, *cpe*)
AN 4279/01^a^	Swedish outbreak 1, meat soup
AN 4280/01	Swedish outbreak 1, meat soup
AN 4281/01	Swedish outbreak 1, meat soup
AN 4282/01	Swedish outbreak 1, meat soup

^a ^Used for sequencing of the 16S rRNA genes

^b ^DNA degraded by endogenous endonucleases

^c ^Isolated from horse no. 5 in the study by Gustafsson *et al*. [40].

^d ^Kindly provided by Prof. P.E Granum, Norwegian School of Veterinary Science, Oslo, Norway

**Table 2:** Distribution of *cpb2 *sequences into phylogenetic clusters

Cluster	GenBank accession numbers
Ia	[GenBank:DQ201544 – GenBank:DQ201553 and GenBank:AJ537530 – GenBank:AJ537533]
Ib	[GenBank:DQ201554 – GenBank:DQ201567, GenBank:AJ537534 – GenBank:AJ537551 and GenBank:AY297455]
Ic	[GenBank:DQ201568 – GenBank:DQ201571, GenBank:AY730632 – GenBank:AY730634 and GenBank:AY730636]
IIa	[GenBank:AY609161 – GenBank:AY609167, GenBank:AY609170 – GenBank:AY609177, GenBank:AY609183, GenBank:AY730630, GenBank:AY730631 and GenBank:AY730635]
IIb	[GenBank:AY609168 – GenBank:AY609169]
IIc	[GenBank:AY609178 – GenBank:AY609182]

**Table 3:** Polymorphisms found in the 16S rRNA genes of eleven *C. perfringens *isolates

Strain	91	162	180	187	203	215	220	235	263	265	383	466	523	591	1006	1245	1263	1401
Strain 13		2G/8A	5T/5C			1T/9C		1T/9C			2G/8A					1T/9C	1T/9A	
CCUG 2035	Y	A	Y	A	Y	Y	C	C	A	C	A	C	G	Y	C	C	A	C
CCUG 44727	Y	A	Y	A	Y	Y	Y	C	A	C	A	C	R	C	C	C	A	C
AN 110/95	Y	A	Y	A	Y	C	Y	Y	A	C	A	Y	G	C	C	Y	W	C
AN 2990/99	C	R	Y	A	Y	C	C	C	A	Y	A	C	G	C	C	Y	A	C
AN 1205/02	Y	A	Y	A	Y	Y	C	C	A	C	A	C	G	C	C	C	W	C
AN 4744/02	C	A	Y	W	C	C	C	C	R	Y	R	C	G	C	Y	Y	A	C
B22	C	A	Y	A	C	C	C	C	A	C	A	C	G	C	C	C	W	C
B26	C	A	Y	A	C	C	C	C	A	C	A	C	G	C	C	C	W	C
B34	Y	A	Y	A	C	C	C	C	A	C	A	C	G	C	C	C	W	C
AN 4279/01	Y	A	T	A	Y	Y	C	C	A	C	A	C	G	C	C	Y	W	Y
455/99	Y	A	T	A	Y	Y	Y	C	A	C	A	C	G	Y	C	Y	A	C

The numbers (91 – 1401) refer to the nucleotide positions of the ten 16S rRNA genes of *C. perfringens*, strain 13. A, C, G, T are standard abbreviations used for the nucleotides and polymorphisms are indicated by the letter code suggested by the Nomenclature Committee of the International Union of Biochemistry (Y = C/T, R = A/G and W = A/T).

**Table 4:** General PCR and sequencing primers used for amplification of 16S rDNA

Designation (direction)^1^	Position^2^	Sequence (5'→3')	Application^3^	Reference
RIT593 (F)	11–35 (U1)	GTT TGA TCC TGG CTC AGG AYD AAC G	PCR	Johansson et al. [36]
kag-001 (R)	1482–1507 (U8)	TAC CTT GTT ACG ACT TMA CCC CAA TC	PCR	This study
RIT583 (F)	13–28 (U1)	TTG ATC CTG GCT CAG G	Seq.	Johansson et al. [36]
RIT584 (F)	334–352 (U2)	CCA RAC TCC TAC GGR AGG C	Seq.	Johansson et al. [36]
RIT631 (R)	519–534 (U3)	ATT ACC GCG GCK GCT G	Seq.	Johansson et al. [36]
RIT538 (F)	800–818 (U4)	GTA GTC CAC GCC GTA AAC G	Seq.	Johansson et al. [36]
kag-011 (R)	922–939 (U5)	CTT GTG CGG GYY CCC GTC	Seq.	This study
kag-006 (F)	1174–1192 (U6)	GGA GGA AGG TRG GRA TGA C	Seq.	This study
kag-002 (R)	1488–1506 (U8)	ACC TTG TTA CGA CTT MAC C	Seq.	This study

^1 ^Forward or reverse (F or R).

^2 ^According to the *E. coli *16S rRNA sequence (universal region no.).

^3 ^PCR or sequencing (Seq.).
