# Supplementary material for: Genetic diversity of Clostridium perfringens type A isolates from animals, food poisoning outbreaks and sludge
Source: BMC Microbiol. 2006 May 31;6:47. doi: 10.1186/1471-2180-6-47 (PMC1513381; doi:10.1186/1471-2180-6-47)
Supplement: Additional File 1 — Consensus alignment of cpb2 clusters [file 1471-2180-6-47-S1.pdf]

```
               1         11         21         31         41        50
               |         |          |          |          |         |
Cluster Ia   AAATATGATC CTAACCAACA ACTGAAATCT TTTGAAATAC TTAATTCACA    50
Cluster Ib   AAATATGATC CTAACCAACA ACTAAAATCT TTTGAAATAC TTAATTCACA    50
Cluster Ic   AAATATGATC CTAACCAACA ACTAAAATCT TTTGAAATAC TTAATTCACA    50
Cluster IIa  AAATATGATC CTAACCAAAA ACTTAAATCA TATGAAATAA CAGkTTCAAG    50
Cluster IIb  AAATATGATC CTAACCAAAA ACTTAAATCA TATGAAATAA CAAGTTCAAG    50
Cluster IIc  AAATATGATC CTAACCAAAA ACTTAAATCA TATGAAATAA CAAGTTCAAG    50

               51        61         71         81         91        100
               |         |          |          |          |         |
Cluster Ia   AAAGAGCGAT AATAAAGAAA TATTTAATGT AAAAACTGAA TTTTTAAATG   100
Cluster Ib   AAAGATTGAT AATAAAGAAA TATTTAATGT AAAAACTGAA TTTATGAATG   100
Cluster Ic   AAAGAGTGAT AATAAAGAAA TATTTAATGT AAAAACTGAA TTTATGAATG   100
Cluster IIa  AAAAATTGAT AATGGyGAAA TTTTTTCTGT GAAAACAGAG TTTTTAAATG   100
Cluster IIb  AAAAATTGAT AATAGTGAAA TTTTTTCTGT GAAAACAGAG TTTTTAAATG   100
Cluster IIc  AAAAATTGAT AATGGTGAAA TTTTTTCTGT GAAAACAGAG TTTTTAAATG   100

               101       111        121        131        141       150
               |         |          |          |          |         |
Cluster Ia   GTGCAATTTA TGATATGGAA TTTACTGTAT CATCTAAAGA TGGAAAATTA   150
Cluster Ib   GTGCAATTTA TGATATGAAA TTTACTGTAT CATCTAAAGA TGGGGAATTA   150
Cluster Ic   GTGCAATTTA TGATATGAAA TTTACTGTAT CATCTAAAGA TGGAAAATTA   150
Cluster IIa  GTGCTATATA CAATATGGAA TTTACAGTAT CATATATTGA TAATAAATTA   150
Cluster IIb  GTGCTATATA TAATATGGAA TTTACAGTAT CATATATTGA TAATAAGTTA   150
Cluster IIc  GTGCTATATA CAATATGGAA TTTACAGTAT CATATATTGA TAATAAGTTA   150

               151       161        171        181        191       200
               |         |          |          |          |         |
Cluster Ia   ATAGTATCTG ATATGGAAAG AACAAAAATT GAGAATGAAG GAAAATATAT   200
Cluster Ib   ATAGTATCTG ACATGGAAAG AACAAAAATT GAGAATGAGG GAAAATATAT   200
Cluster Ic   ATAGTATCTG ACATGGAAAG AACAAAAATT GAAAATGAGG GAAAATATAT   200
Cluster IIa  ATGGTAAGTA ATATGAATAG AATATCAATA GTAAATGAAG GTAAATrTAT   200
Cluster IIb  ATGGTAAGTG ATATGAATAG AATAGCAATA ATAAATGAAG GTAAACGTAT   200
Cluster IIc  ATGGTAAGTG ATATGAATAG AACAGCAATA ATAAATGAAG GTAAACGTAT   200

               201       211        221        231        241       250
               |         |          |          |          |         |
Cluster Ia   TTTAACACCA TCATTTAGAA CTCAAGTTTG TACATGGGAT GATGAACTAG   250
Cluster Ib   TTTAACACCA TCATTTAGAA CTCAAGTTTG TACATGGGAT GATGAATTAT   250
Cluster Ic   TTTAACACCA TCATTTAGAA CTCAAGTTTG TACATGGGAT GATGAATTAT   250
Cluster IIa  TCCTACACCA AGTTTCAGAA CTCAAGTTTG TACATGGGAT GACGAATTAA   250
Cluster IIb  TCCTACACCA AGTTTCAGAA CTCAAGTTTG TACATGGGAT GATGAATTAA   250
Cluster IIc  TCCTACGCCA AGTTACAGAA CTCAAGTTTG TACATGGGAT GACGAATTAA   250

               251       261        271        281        291       300
               |         |          |          |          |         |
Cluster Ia   CACAAGCAAT TGGGGGAGTT TATCCACAAA CATATTCTGA TAGATTTACA   300
Cluster Ib   CACAATCAAT TGGGGGAGTT GATCCAAAAA CATATTCTAC TAGATTTACA   300
Cluster Ic   CACAATCAAT TkGGGGAGTT GATCCAAAAA CATATTCTAC TAGATTTACA   300
Cluster IIa  GTCAATATAT TGGAGACGCT GTTAGTTTTA CACGTTCTAG TAAATTTCAA   300
Cluster IIb  GTCAATATAT TGGAGACGCT GTTAGTTTTA CACGTTCTAG TAAATTTCAA   300
Cluster IIc  GCCAATATAT TGGAGATGCT GTTAGCTATA CACGTTCTGA TAAATTCAAA   300

                 301       311        321        331        341       350
               |         |          |          |          |         |
Cluster Ia   TATTATGCAG ATAATATATT ATTAAACTTC AGACAATATG CAACTTCAGG   350
Cluster Ib   TATTATGCAG ACAATATATT ATTAAACTTT AGACAATATG CAACTTCAGG   350
Cluster Ic   TATTATGCAG ACAATATATT ATTAAACTTT AGACAATATG CAACwTCAGG   350
Cluster IIa  TATAGTTCTA ATACGATTAC ATTAAACTTT AGACAATATG CAACTTCTGG   350
Cluster IIb  TATAGCTCTA ATACTATTAC ATTAAACTTT AGACAATATG CAACTTCTGG   350
Cluster IIc  TATAGCTCTA ATACTATTAC ATTAAACTTT AGACAATATG CAACTTCTGG   350

               351       361        371        381        391       400
               |         |          |          |          |         |
Cluster Ia   TTCAAGAGAT TTAAAAGTAG AATATAGTGT TGTAGATCAT TGGATGTGGA   400
Cluster Ib   TTCAAGAGAT TTAAAAGTAG AATATAGTGT TGTAGATCAT TGGTTATGGG   400
Cluster Ic   TTCAAGAGAT TTAAAAGTAG AATATAGTGT TGTAGATCAT TGGGTATGGG   400
Cluster IIa  ATCAAGATCC TTAAAGGTAA AATAmAGTGT AGTAGACCAT TGGATGTGGG   400
Cluster IIb  ATCAAGATCA TTAAAGGTAA AATACAGTGT AGTAGACCAT TGGATTTGGG   400
Cluster IIc  ATCAAGATCA TTAAAGGTAA AATACAGTAT AGTAGACCAT TGGCTTTGGG   400

               401       411        421
               |         |          |
Cluster Ia   AAGATGATGT TAAAGCTTCT CAAAT                              425
Cluster Ib   GAGATGATGT TAAAGCTTCT CAAAT                              425
Cluster Ic   GAGATGATGT TAAAGCTTCT CAAAT                              425
Cluster IIa  GGGATGACAT TAGAGCTTCT CAATG                              425
Cluster IIb  GGGATGACAT TAGAGCTTCT CAATG                              425
Cluster IIc  GTGATGACAT TAGAGCTTCT CAATG                              425
```
